# Validation of subjective manual palpation using objective physiological recordings of the cranial rhythmic impulse during osteopathic manipulative intervention

**DOI:** 10.1038/s41598-023-33644-8

**Published:** 2023-04-24

**Authors:** Holger Pelz, Gero Müller, Micha Keller, Klaus Mathiak, Johannes Mayer, Stefan Borik, Volker Perlitz

**Affiliations:** 1 Deutsche Gesellschaft für Osteopathische Medizin e.V., St.-Petri-Platz 5, 21614 Buxtehude, Germany; 2 Department of Psychiatry, Psychotherapy and Psychosomatics, Faculty of Medicine, RWTH Aachen, Aachen, Germany; 3 JARA, Translational Brain Medicine, Aachen, Germany; 4 Department of Electromagnetic and Biomedical Engineering, Faculty of Electrical Engineering and Information Technology, University of Zilina, Zilina, Slovakia; 5 Simplana GmbH, Neuenhoferweg 25, 52074 Aachen, Germany

**Keywords:** Medical research, Peripheral nervous system

## Abstract

Intermediate (IM) band physiology in skin blood flow exhibits parallels with the primary respiratory mechanism (PRM) or cranial rhythmic impulse (CRI), controversial concepts of osteopathy in the cranial field (OCF). Owing to inconsistent manual palpation results, validity of evidence of PRM/CRI activity has been questionable. We therefore tried to validate manual palpation combining instrumented tracking and algorithmic objectivation of frequencies, amplitudes, and phases. Using a standard OCF intervention, cranial vault hold (CVH), two OCF experts palpated and digitally marked CRI frequencies in 25 healthy adults. Autonomic nervous system (ANS) activity in low frequency (LF) and IM band in photoplethysmographic (PPG) forehead skin recordings was probed with momentary frequency of highest amplitude (MFHA) and wavelet amplitude spectra (WAS) in examiners and participants. Palpation errors and frequency expectation bias during CVH were analyzed for phases of MFHA and CRI. Palpated CRI frequencies (0.05–0.08 Hz) correlated highly with mean MFHA frequencies with 1:1 ratio in 77% of participants (LF-responders; 0.072 Hz) and with 2:1 ratio in 23% of participants (IM-responders; 0.147 Hz). WAS analysis in both groups revealed integer number (harmonic) waves in (very) low and IM bands in > 98% of palpated intervals. Phase analyses in participants and examiners suggested synchronization between MFHA and CRI in a subset of LF-responders. IM band physiology in forehead PPG may offer a sensible physiological correlate of palpated CRI activity. Possible coordination or synchronization effects with additional physiological signals and between examiners and participants should be investigated in future studies.

## Introduction

Osteopathic manipulative treatment (OMT) focuses on enhancing the body’s self-regulation capacity by influencing autonomic nervous system (ANS) activity. Increasing evidence of heart rate variability (HRV) research in healthy non-symptomatic participants suggests OMT may have a positive effect by modulating the parasympathetic branch^1–6^. Osteopathy in the cranial field (OCF), however, rests on concepts which are still a matter of controversy. This concerns the cranial rhythmic impulse (CRI) and the primary respiratory rhythm (PRM). The former denotes rhythmic movements on the cranial level, whereas the latter allocates such movements to the remaining body. The diagnosis and therapy of somatic dysfunctions rest on modulations of the PRM/CRI since both physiological phenomena are associated with autonomic nervous system (ANS) activity. Previous studies on CRI/PRM dynamics have identified such oscillations as Traube-Hering (Mayer) (TH or THM; resp.) waves with a frequency centered at approx. 0.1 Hz^7–11^.

Technical approaches investigating the physiology underlying the CRI were promising yet inconclusive due to their poor match with palpation^12^. Association of CRI/PRM with TH waves in blood pressure, heart rate, respiration, and skin blood flow have been suggested as physiological correlate for the palpation of the CRI/PRM within all parts of the body. However, palpated CRI rates differed frequently by a factor 2 from rates recorded using technical instrumentation^10^. The CRI/PRM thus remains an enigmatic phenomenon, which poses many open questions.

Hitherto, ANS activity relevant for cardiovascular and respiratory systems has been widely accepted to appear as a low frequency (LF, 0.05–0.15 Hz) and a high frequency (HF, 0.15–0.4 Hz) band. This concept and that of TH waves supposedly accounting for the CRI/PRM have recently been challenged suggesting the CRI/PRM as a manifestation of ‘0.15 Hz rhythm band’ physiology^13^, also referred to as intermediate (IM) band. Activity in the ‘0.15 Hz frequency band’ has been shown to emerge in various peripheral systems in humans during hypnoid relaxation^14–17^. These authors had hypothesized that the emergence of the ‘0.15 Hz rhythm band’ in humans corresponds with findings in canine unspecific neurons in the reticular formation of the lower brainstem termed the ‘retR’^16,18^. Recent functional magnetic resonance imaging (fMRI) studies suggest IM band fluctuations in humans also originate in the brainstem^19^. Furthermore, IM band activity in heart rate variability (HRV) and respiration was associated with blood oxygen level-dependent (BOLD) activity in an interoceptive processing network^20^. It is of note, that recent suggestions view interoception as a key to a neurological model in osteopathy^21^.

The frequency range of the IM band (0.15 Hz ± 0.03 Hz), the formation of beat oscillations, and integer number phase-synchronization with respiration, HRV, and blood pressure^16^ are all well in keeping with currently held notions on explaining the origin of the CRI/PRM. Moreover, upper or lower harmonic waves of IM primary band^17^ may well account for reported regular variations in palpated CRI rates, a still puzzling phenomenon of the CRI/PRM^10^. The possibility of lower harmonic waves as a source of rhythmic activity has previously been discussed by osteopathic researchers^11^.

The hypothesis put forth by Pelz^13^ has gained further support from current results of our research group. In a larger group a standard OCF treatment, cranial vault hold (CVH), triggered an immediate decline in forehead skin blood oscillations from activity at 0.12 Hz (IM band activity) during control conditions (eyes open, eyes closed) to stable activity in the LF band at 0.07 Hz. These oscillations may represent lower harmonic waves of the primary IM band^22^. A smaller subset of participants remained at primary IM band frequencies. Open questions concern the validation of palpated events with ANS related rhythmic activity in skin blood flow waves. A previous study demonstrated a poor interrater reliability, which put the validity of OMT further into question^23^. Also, the observation that CRI rates palpated by unexperienced examiners were found to be consistently above those palpated by more experienced examiners put the entire concept further into question^24^. Yet another questionable concept concerns the current OMT model used to describe palpated events as ‘flexion’ and ‘extension’ events, supposed to account for one complete CRI cycle. However, one CRI cycle does not consistently amount to supposedly complete rates (1:1 ratio) attained by instrumented recordings as higher palpated rates (2:1 ratio) have also been reported^10^.

To resolve these questions concerning the validity of the CRI/PRM concept of OCF, we designed a hardware-software approach, which combined a Bluetooth-equipped footswitch to mark palpated rhythmic activity with semi-linear high temporal resolution algorithms for the analyses of frequency, amplitudes, and phases. This approach should allow to reliably test the validity of ANS physiological activity and concomitant palpated CRI rhythmic activity during CVH, a specific OCF intervention.

Employing these techniques, we tested the following hypotheses:

- (1) Time intervals palpated at the skull and marked with the footswitch match CRI *rates* reported in the literature for experienced examiners;
- (2) Palpated CRI *rates* are equivalent to autonomic nervous system (ANS) rhythmic activity in photoplethysmographic (PPG) forehead skin recordings in frequencies of IM-band physiology (0.12–0.18 Hz) and their corresponding lower harmonic waves (0.06–0.09 Hz);
- (3) Wavelet amplitude spectra (WAS) detect primary IM-band and its harmonic waves of lower amplitude responsible for rhythmic activity confounding palpation results;
- (4) Phasic footswitch responses contain information on palpated CRI ANS related activity which is reflected also by dynamics of momentary frequency of highest amplitude (MFHA);
- (5) Phases of MFHA correlate with manually palpated ‘flexion' and ‘extension’.

## Methods

### Participants

A convenience sample of twenty-five healthy adults (14 female, age 42.4 (± 13.1) years) was recruited by the investigators through word of mouth. Five participants completed two measurements at an interval of 1 week, resulting in a total of 31 measurements. One measurement had to be excluded, as palpation records were faulty. All participants were non-smokers, none had any OMT in the preceding three months. Exclusion criteria were mental health symptomatology, a history of or acute neurological or cardiovascular disorders, current use of psychoactive medication as well as high-performance sport. Furthermore, participants were asked to abstain from caffeine for 4 h and alcohol consumption for 48 h prior to testing. The experiment was conducted according to the Code of Ethics of the World Medical Association (Declaration of Helsinki, 2008). All participants provided written, informed consent, and all protocols were approved by the Institutional Review Board of the state of Lower-Saxony/Germany (EK vote from 04/03/2017).

### Examiners

Two DGOM-certified osteopaths participated in the study as examiners. At the time of the study, examiner A had been in private practice for 34 years and was also teaching OCF. Examiner A estimated that 60% of patients under his care would undergo at least some cranial treatment, whereas cranial treatment would be the major treatment regimen for approximately 50% of patients. Examiner B had been in private practice for 25 years and was also teaching OCF. Examiner B estimated that approximately 80% of patients under his care would receive at least some cranial treatment whereas this would be the major treatment for approximately 60% of patients.

### Study design

**Figure 1:**
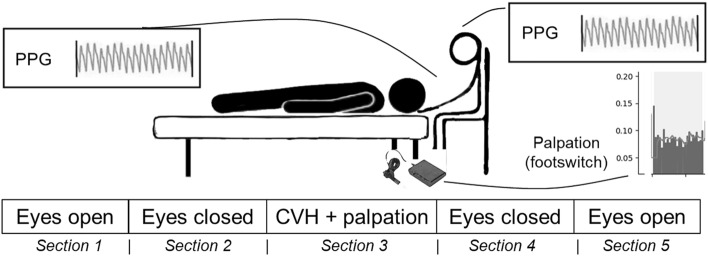
***Experimental paradigm.*** Each session was comprised of 5 sections, lasting 300/330 seconds each. Section 1 and 5 (eyes open) as well as section 2 and 4 (eyes closed) were performed “hands-off”. Section 3 was “hands-on” during which examiners applied the cranial vault hold (CVH), recording palpation intervals by operating the Bluetooth footswitch. During the entire session, forehead skin blood oscillations (photoplethysmography) were recorded for participant and examiner.

The study used a double-blind design with examiners as well as participants being naïve to data recording and data analysis performed offline. A between-subjects design was used, however, all participants analyzed in the current manuscript had been allocated to the cranial vault hold (CVH) intervention employed to palpate triggered CRI activity. Performed at comfortable room temperature, experimental sessions consisted of five consecutive sections (initially 300 s, but extended to 330 s after the first measurements to account for artifacts due to section transitions). These additional sections were clipped from all signals prior to analysis. CVH sections were braced by eyes-open (EO) and eyes-closed (EC) sections serving as within-subject control conditions. During CVH eyes were kept closed as well. Thus, the final order was: EO, EC, EC + CVH, EC’, EO’. The two resting state phases were “hands-off” during which the physician’s hands had no contact with the participant. The third phase employing CVH was the only “hands-on” phase (see also Fig. 1). As the current investigation focused on physiological correlates of palpation sensations, only the CVH section was analyzed.

CVH is a standardized osteopathic hands-on technique which incites defined cranial regions and cranial bones at very low pressure on the scalp. Examiners utilized commonly agreed techniques to execute CVH^25^. Upon recognition of an arriving tide (in OCF commonly denoted as extension) the footswitch was pressed, and upon subsiding of the tide (denoted as flexion) the footswitch was released. Therefore, the interval between two consecutive footswitch operations marks the extension part of the CRI. This procedure is in line with most CRI trials that recorded laser Doppler flowmetry (LDF) data of skin blood perfusion^9^. PPG recordings used a reflective PPG probe placed on the glabella forehead region of participant and examiner. The examiner was seated behind the head of the participant who remained in a supine position.

### Instrumentation

**Figure 2:**
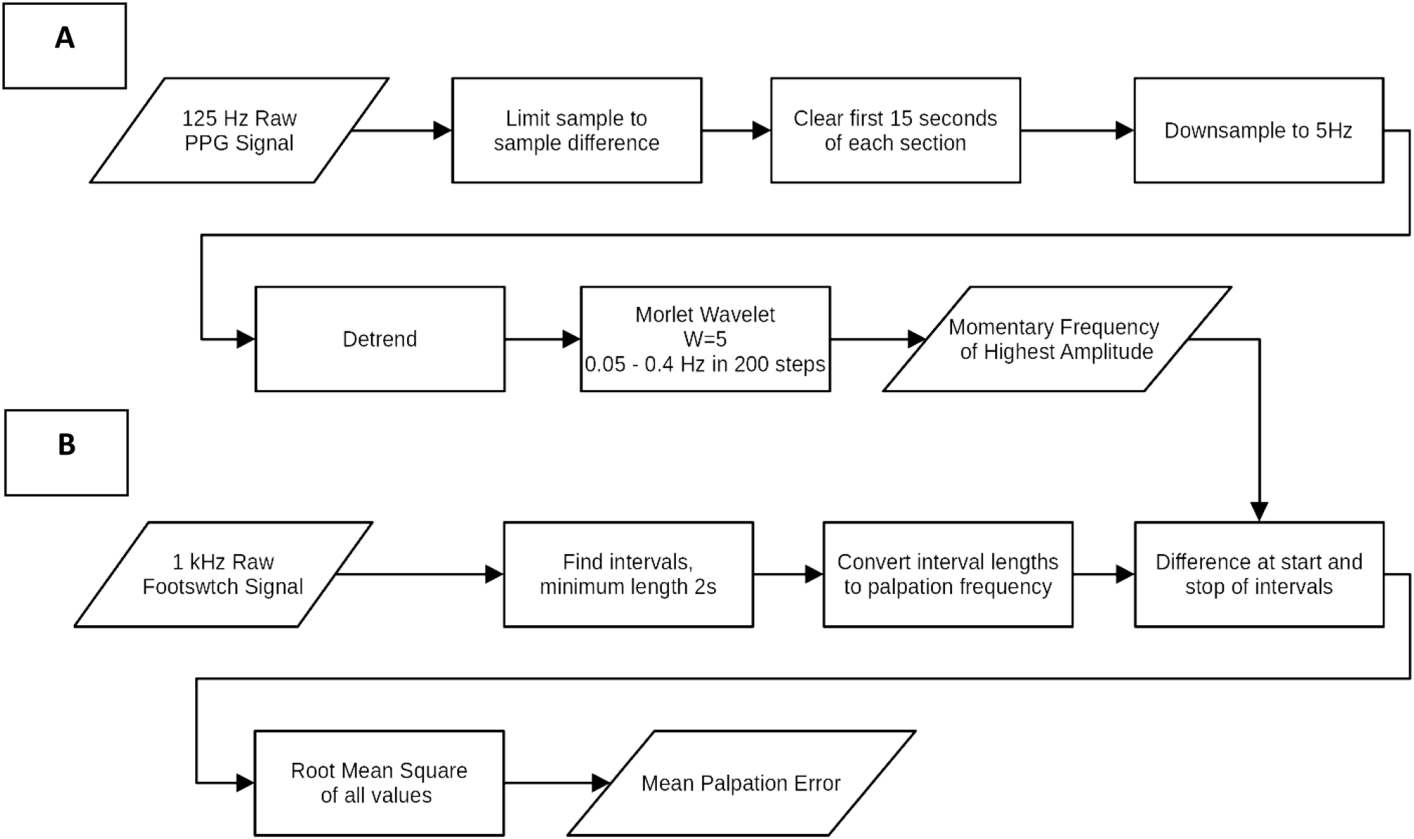
***Data processing and analysis steps.*** (**A**) Photoplethysmography data was recorded using a sampling frequency of 125 Hz. Next, artifacts were removed by detection of large jumps in signal amplitude (limited to 3·*σ*) and removal of the first 15 s of each section due to proneness to movement artifacts. To improve further data processing, the signal was subsampled to 5 Hz and detrended. The preprocessed signal was then converted to a time–frequency distribution using the continuous wavelet transformation and a Morlet wavelet (with parameter *σ* = 5). The frequency ranged from 0.05 to 0.4 Hz and was sampled in 200 steps. Finally for each time step the largest frequency was extracted. (**B**) Extension intervals of palpated CRI activity in human scalp perfusion were marked with a Bluetooth equipped footswitch. The time series generated had a 1 kHz resolution to allow for high resolution analysis. In order to compute the lengths of complete CRI cycles, palpated durations of extension (in [s]) intervals were multiplied by a factor 2. These CRI durations (in [s]) were then converted to physical frequencies [Hz]. The frequencies computed this way replaced the original extension intervals on the x-axis, enabling comparison with frequencies recorded in skin perfusion.

A commercially available footswitch was in-house modified to be able to track palpation sensations of CRI activity using a Bluetooth module. Prior to the recordings used for the study, examiners practiced sufficiently to get acquainted with the use of the footswitch. The PPG device used a standard Osram SFH7060 PG sensor able to emit red, infrared, and green light and simultaneously detect signals using a built-in photodiode. The data from this sensor are further processed with a microcontroller firmware which recorded the reflected red-light signals at 660 nm at a sample rate of 125 Hz since we focused on autonomic nervous system oscillations < 0.4 Hz only (see Fig. 2). The recorded data was synchronized using timestamps. All sensors (participant PPG, examiner PPG, and examiner footswitch) were time-synchronized in the beginning of each measurement with a relative difference of less than 10 ms. The synchronized data stream was transmitted to a local computer using Bluetooth 4.0 protocol, and the entire measurement was uploaded to a private cloud for future offline processing.

### Grouping LF- and IM-responders

In a parallel publication^22^  we reported on differential physiological responses in LF, IM, and HF activity during CVH computed from PPG data. Submitted to a *K*-means cluster analysis^26^ participants were clustered into different groups based on their physiological responses during CVH. This resulted in one group showing a sudden decline from 0.14 (primary IM band activity) to 0.072 Hz (LF activity), whereas a second group remained in IM mode. The former was therefore referred to as LF-responders, and the latter referred to as IM-responders.

### Data processing and statistical analyses

#### Serial analyses and preparation of PPG data

Processing of PPG data was performed in Python using Numpy^27^, SciPy^28^, and Matplotlib^29^ libraries. Processing routines used for serial analysis of the entire study sample followed data analyses detailed in Fig. 2. Datasets with obvious sections of artefacts were excluded from further data processing. Of the remaining 31 datasets, the number of corrected data of affected time series was < 1%. Processing included analyses of time–frequency distributions for the various frequency bands and conversion of these compressed time series to aggregated values used to compute statistics. Continuous wavelet transformation (CWT) using Morlet wavelets were computed and then reduced to 2D-time series of momentary frequencies of highest amplitude (MFHA, also known as time–frequency ridge), used to identify objective physiological correlates of palpated CRI activity. MFHA and CRI data was imported to the Statistical Package for the Social Sciences (SPSS), version 27 (SPSS Inc., Chicago, IL, USA) for statistical analyses. A *p*-value of < 0.05 was considered statistically significant. Descriptive data of MFHA, CRI, and their deviations was computed. Furthermore, a more precise estimate of deviations of palpations from MFHA was obtained by computing the root mean square of differences between MFHA and CRI. Pearson’s *r* correlations were computed for LF- and IM-responders. A linear mixed model was run using mean CRI frequency as dependent variable and mean MFHA frequency, response (LF vs. IM responders), examiner (A vs. B), timepoint (1 vs. 2) as fixed effects. Participant was added as random effect, including the random intercept. The model specification was as follows: Mean CRI frequency ~ mean MFHA frequency + response + examiner + timepoint + (1|Participants). Significance was calculated based on Satterthwaite’s method, estimating degrees of freedom and generating *p*-values.

#### Wavelet amplitude spectra

MFHA analysis may discard important physiological information on frequencies of lower amplitude and may thereby account for a misleading display of frequencies. Such frequencies of lower amplitude are of particular relevance in our context since they may represent lower or upper harmonic waves of IM or LF band activity. To gain a better understanding of frequencies of PPG data and palpated extension intervals, we used multiple Gaussians to fit the wavelet amplitude spectrum (WAS), since the wavelet response is falling off with the distance to the underlying frequency:

1 $$g\left(f\right)=\sum {a}_{i}\cdot {e}^{\frac{-{\left(f-{b}_{i}\right)}^{2}}{2{c}_{i}^{2}}},$$

where *f* is the frequency, *a* is the height of the curve's peak, *b* is the position of the center of the peak, and *c* is the width of the curve. Since the number of dominant frequencies may be different at each point in time, we repeated the fit with *n* = 1 − 6 Gaussians and selected the function which described the WAS best (see Fig. 5). Several examples of amplitude-frequency distributions were examined, which were considered to be representative for the majority of data.

#### Phase computations and analyses

In an exploratory analysis, phases of palpated frequencies and MFHA at the time of push (start) and release (stop) of the footswitch were computed for examiners and participants. This was done to exclude a possible ‘frequency bias’ towards expected frequencies unrelated to palpated frequencies. Histograms of phases of palpated sections as well as MFHA were computed to examine phase occurrences. Palpation errors (deviation of MFHA and CRI frequency for each palpated interval) were plotted against phases of CRI or MFHA. Furthermore, to probe co-occurrence of phases in CRI and MFHA, scatterplots were created for both parameters for participants as well as examiners. Scatterplots were investigated for certain patterns such as grouping of CRI and MFHA phases at certain phases. Pearson’s *r* correlations were computed for CRI and MFHA phases and independent *t*-tests compared mean correlation coefficients between participants and examiners in each group (LF-responders, IM-responders).

## Results

### Frequencies of palpated waves and momentary frequency of highest amplitude

**Figure 3:**
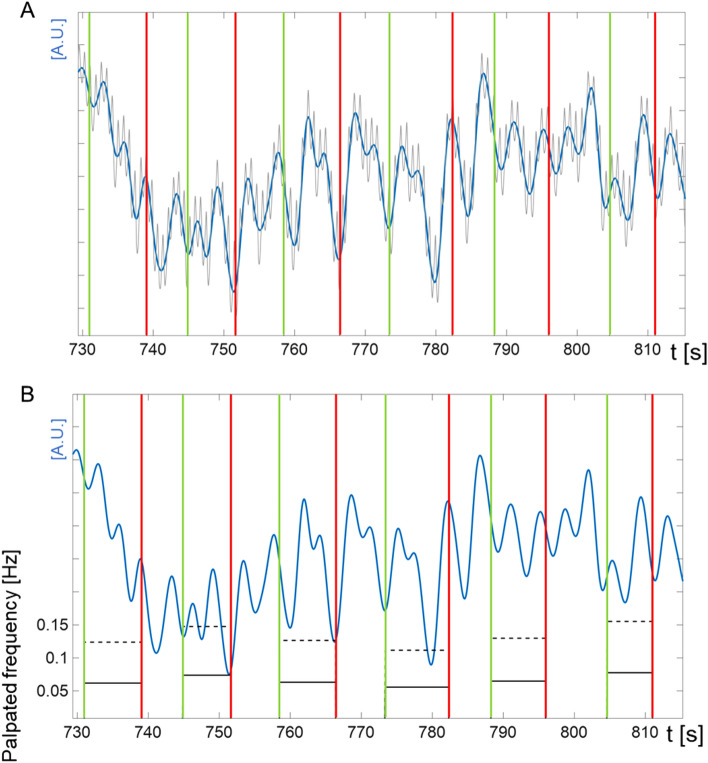
***Exemplary section from cranial vault hold of participant A14.*** (**A**) upper panel: Grey: lowpass-filtered (5 Hz) PPG signal; blue: slow PPG oscillations (lowpass-filtered at 0.5 Hz); vertical lines: palpated extension sections of cranial rhythmic impulse (CRI) with start (green; pressing of footswitch) and stop (red; releasing of footswitch). (**B**) lower panel: Grey: lowpass-filtered (5 Hz) PPG signal; blue: slow PPG oscillations (lowpass-filtered at 0.5 Hz); vertical lines: palpated extension sections of cranial rhythmic impulse with start (green; pressing of footswitch) and stop (red; releasing of footswitch); solid horizontal black lines: computed extension frequency; dashed horizontal black lines: computed CRI. The CRI activity was consistently palpated between 0.05 and 0.1 Hz.

Examiners palpated 377 extension intervals in LF-responders who responded to CVH (N = 24) with a sudden decline from 0.14 (primary IM band activity) to 0.072 Hz (LF activity), and 124 extension intervals in IM-responders (N = 7) that remained in IM mode during CVH. In a first step of signal analysis, the palpated ’extension’ time intervals, calculated extension frequencies, and CRI frequencies computed thereof were superimposed with lowpass filtered (5 Hz) PPG data (Fig. 3A and B). This showed that palpated extension intervals captured waves in PPG signals at IM band frequencies between 0.12 and 0.16 Hz (≡ 7.2–9.6 cpm; see e.g., recording ‘participant A14’ in Fig. 3). Dividing extension rates by 2 yielded CRI rates (i.e., a complete cycle of extension and flexion) in the LF band between 0.06 and 0.07 Hz (≡ 3.6–4.2 cpm). Such rates are in agreement with rates reported in the literature for experienced examiners^10,24^.

**Figure 4:**
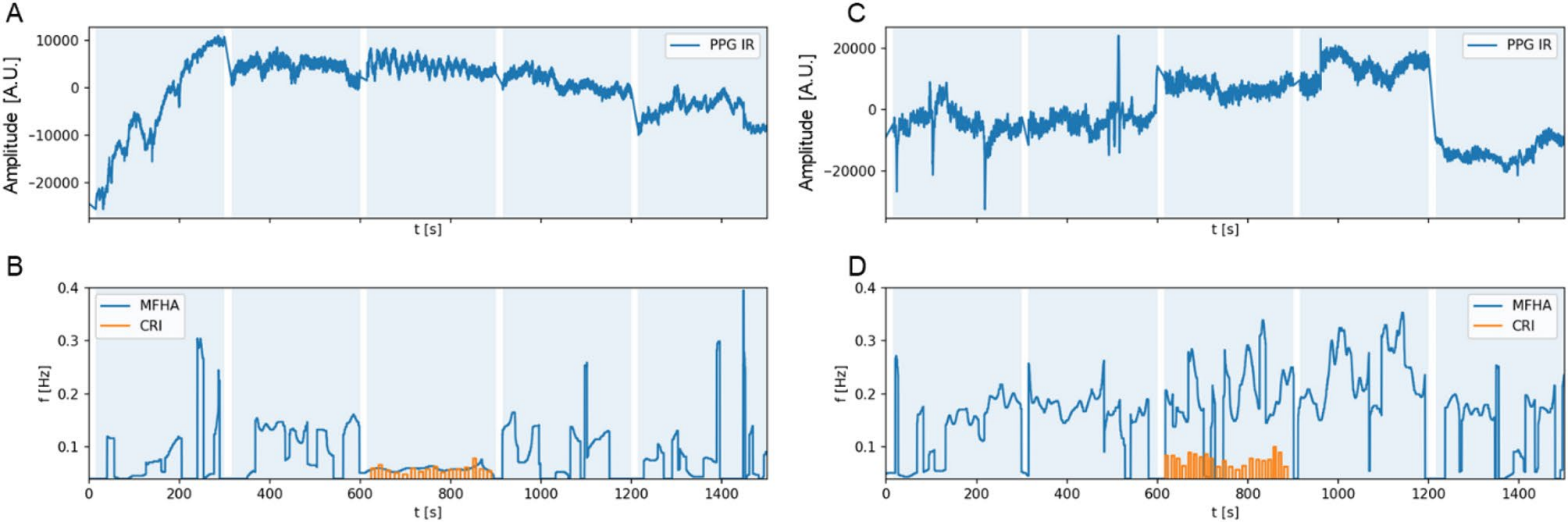
Raw forehead photoplethysmography (PPG) data of (**A**) participant A5 and (**C**) participant A14: consecutive recordings of five 300 s blocks. White bars indicate extension of 15 s in the beginning and end of each section accounting for transition effects. X-axis: time in [s], y-axis: arbitrary units [A.U.]. Note distinct beat (or resonance or spindle) waves in response to CVH in (**A**). **(B)** participant A5**:** momentary frequency of highest amplitude (MFHA, blue line) obtained from Morlet wavelet time frequency distributions of forehead skin PPG recordings. During CVH the plot shows stable low frequency band activity at approx. 0.06 Hz. Palpated CRI intervals converted to frequency equivalents [Hz] (orange graphs) exhibit high agreement with PPG data. This is supported by a high correlation (*r* = .72) with MFHA. (**D**) participant A14: MFHA (blue line) of forehead skin PPG recording. During CVH, the plot shows IM-band activity oscillating at approx. 0.15 Hz with transient high frequency (HF) bursts and transient low-frequency episodes at 0.06 Hz, followed by changes between IM-band and HF-band activity. Palpated extension intervals [Hz] (orange graphs) exhibit poor, if any agreement with MFHA which is reflected by large deviation of palpation and MFHA. Order of protocol: 1. eyes open (EO), 2. eyes closed (EC), 3. EC + CVH, 4. EC’, 5. EO’.

For further visual inspection, MFHA of PPG forehead skin blood flow were superimposed on CRI rates during CVH (see Fig. 4A–D for two representative subjects). The raw PPG data of participant A5 (LF-responder) showed prominent large waves, also known as beat, resonance, or spindle waves at stable low oscillating LF-band activity at 0.07 Hz (Fig. 4A). Superimposing this graph with computed CRI rates exhibited a distinct fit (Fig. 4B). Raw PPG data of participant A14 (IM-responder) (Fig. 4C and D) showed no beat or spindle waves during CVH. Here, MFHA exhibited unstable IM band activity with two short lived episodes of LF activity and hardly any fit between MFHA and computed CRI activity. Table 1 lists computed mean CRI activity, corresponding MFHA rates, and differences between these (in [Hz]) for the two subsets of participants. For LF-responders, independent *t*-tests showed a non-significant difference between examiner A and B for mean MFHA (*t*(21) = 2.0, *p* = 0.06) and a significant difference for mean palpation frequency (*t*(21) = 2.1, *p* = 0.05). However, there was a non-significant difference between examiner A and B for “MFHA-Palpation deviations” (*t*(21) = 0.7, *p* = 0.44). In LF-responders, the computed CRI frequencies and MFHA frequencies were at a ratio of 1:1. Here, the absolute numbers of mean MFHA matched the mean of computed absolute CRI frequencies in most cases (*M*_MFHA_ = 0.071 Hz; *M*_CRI_ = 0.066 Hz). In IM-responders this ratio was 1:2 since absolute numbers of mean MFHA and the mean of computed absolute CRI rates differed by a factor 2 (*M*_MFHA_ = 0.147 Hz; *M*_CRI_ = 0.068 Hz). Correlation analysis of mean MFHA and mean computed CRI frequencies showed high correlations in LF-responders (*r* = 0.72, *p* < 0.001) as well as in IM-responders (*r* = 0.83, *p* < 0.05). These exploratory correlation analyses encompassed within- and between-subjects variance since 5 of the participants were palpated twice. Nevertheless in a mixed linear model, the association between mean MFHA and mean CRI frequencies remained significant when considering the noise structure (*F*(1, 13.65) = 35.8, *p* < 0.001). Furthermore, the effect of different examiners (*F*(1, 22.2) = 0.98, *p* = 0.33) as well as the effect of the repeated measure (*F*(1, 6.3) = 2.2, *p* = 0.18) did not reach significance.

**Table 1:** Frequency of CRI activity and momentary frequency of highest amplitude (MFHA) and differences for examiner A and B, and LF- and IM-responders.

Examiner	Group	Participant	CRI activity (Mean ± Std [in Hz])	MFHA(Mean ± Std [in Hz])	Difference (MFHA–Palp. [in Hz])
A	LF-responder	A1	0.082 (± 0.009)	0.108 (± 0.053)	0.027
		A2 (1st)	0.087 (± 0.013)	0.101 (± 0.034)	0.014
		A3 (1st)	0.062 (± 0.015)	0.087 (± 0.035)	0.024
		A4 (1st)	0.057 (± 0.005)	0.058 (± 0.005)	0.001
		A4 (2nd)	0.058 (± 0.015)	0.062 (± 0.022)	0.004
		A5	0.059 (± 0.008)	0.058 (± 0.004)	− 0.001
		A6	0.075 (± 0.008)	0.077 (± 0.027)	0.002
		A7	0.087 (± 0.015)	0.086 (± 0.006)	− 0.001
		A8	0.075 (± 0.009)	0.090 (± 0.029)	0.014
		A9	0.067 (± 0.012)	0.063 (± 0.004)	− 0.004
		A10	0.073 (± 0.008)	0.071 (± 0.007)	− 0.002
		A11	0.060 (± .011)	0.064 (± 0.006)	0.004
		A12	0.063 (± 0.010)	0.068 (± 0.027)	0.006
		***All***	0.070 (± 0.011)	0.076 (± 0.016)	0.007
	IM-responder	A2 (2nd)	0.054 (± 0.021)	0.097 (± 0.038)	0.043
		A3 (2nd)	0.045 (±0 .008)	0.118 (± 0.051)	0.074
		A13	0.069 (± 0.007)	0.130 (± 0.064)	0.061
		A14	0.077 (± 0.011)	0.198 (± 0.061)	0.121
		A15	0.086 (± 0.010)	0.157 (± 0.079)	0.072
		A16	0.094 (± 0.010)	0.204 (± 0.079)	0.111
		***All***	0.070 (± 0.019)	0.151 (± 0.044)	0.080
B	LF-responder	B1	0.059 (± 0.008)	0.057 (± 0.008)	− 0.002
		B2	0.060 (± 0.021)	0.057 (± 0.021)	− 0.003
		B3	0.063 (± 0.011)	0.083 (± 0.011)	0.021
		B4	0.060 (± 0.007)	0.056 (± 0.007)	− 0.004
		B5	0.053 (± 0.008)	0.052 (± 0.008)	− 0.001
		B6 (2nd)	0.050 (± 0.009)	0.058 (± 0.009)	0.008
		B6 (1st)	0.058 (± 0.008)	0.053 (± 0.008)	− 0.004
		B7 (2nd)	0.050 (± 0.008)	0.070 (± 0.008)	0.020
		B8	0.081 (± 0.050)	0.061 (± 0.005)	− 0.020
		B9	0.072 (± 0.030)	0.089 (± 0.030)	0.017
		***All***	0.060 (± 0.010)	0.064 (± 0.013)	0.003
	IM-responder	B7 (1st)	0.051 (± 0.013)	0.127 (± 0.013)	0.077

CRI activity was computed from palpated extension time intervals.

Ratios of mean CRI/MFHA for non-responders ranged from 0.38 to 0.55, which yielded an average of 0.46 (approx. 1:2 ratio).

**Figure 5:**
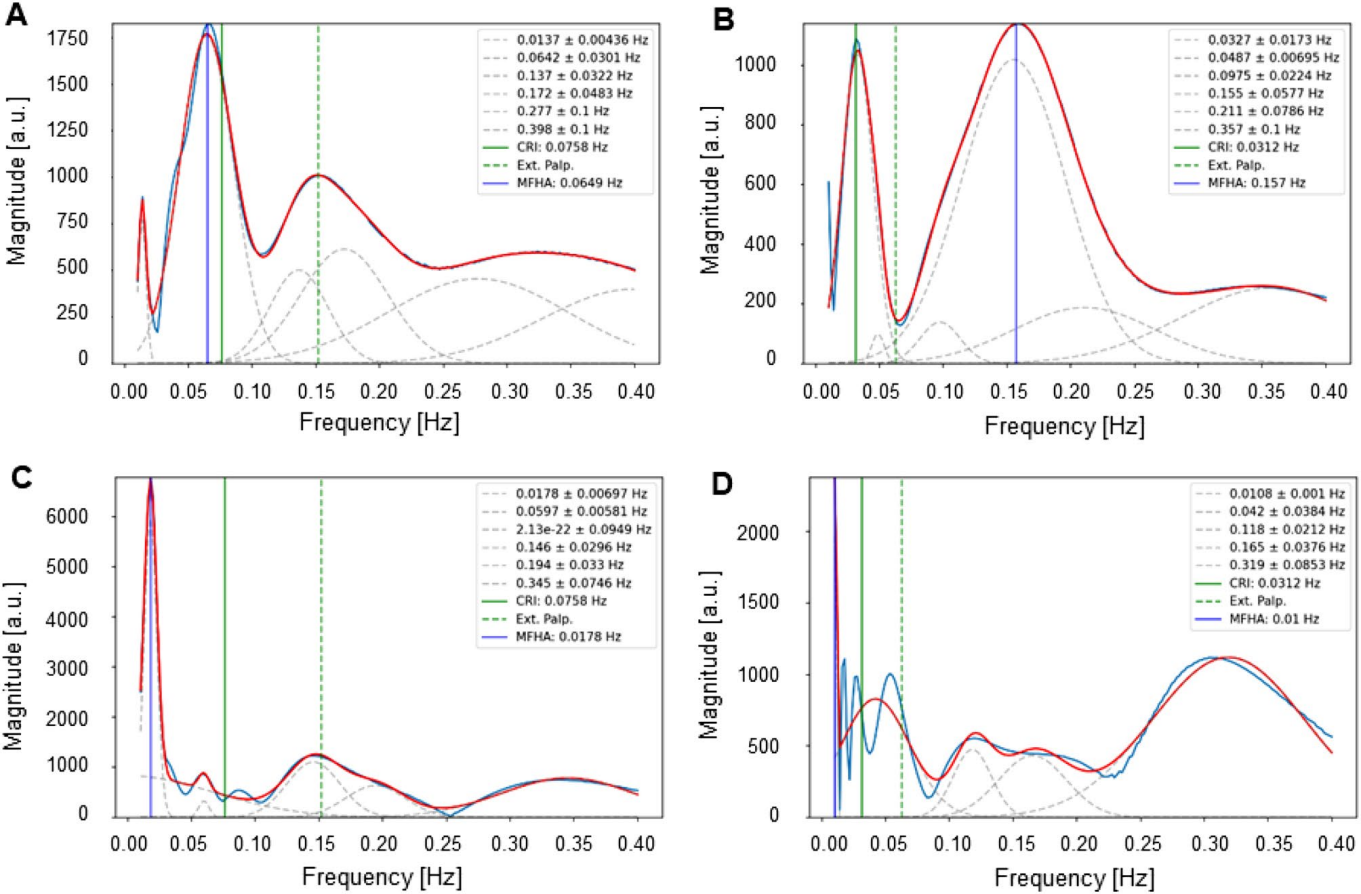
Testing amplitude-frequency distribution. Blue line: momentary frequency of highest amplitude (MFHA) computed from wavelet amplitude spectrum (WAS); green dashed line: palpated extension section; green solid line: computed CRI activity; red line: sum of multiple Gaussian fit; grey dashed lines: multiple Gaussian fit. X-axis: frequency [in Hz], y-axis: arbitrary units. (**A**) The first recorded palpation interval of participant A2 (1st measurement; LF-responder). MFHA shows a dominant wave at 0.065 Hz. The palpated extension section was at 0.154 Hz while the computed CRI activity was at 0.076 Hz (≡ 4.5 cpm), which corresponds well with a prominent wave centered at 0.065 Hz. (**B**) Ninth recorded palpation interval of participant A2 (2nd measurement; IM-responder). MFHA with dominant wave at 0.157 Hz. Palpated extension section at 0.064 Hz between the two dominant peaks of MFHA and VLF activity. CRI activity at 0.035 Hz (≡ 2.09 cpm). (**C**) MFHA of examiner A’s PPG during the first recorded palpation interval of participant A2 (1st measurement; LF-responder). Identical MFHA activity in participant and examiner palpated as an extension section at 0.154 Hz. CRI activity at 0.076 Hz (≡ 4.5 cpm). There was no wave corresponding with this CRI activity. (**D**) MFHA of examiner during palpation of the ninth recorded interval of participant A2 (2nd measurement; IM-responder). MFHA with a slightly dominant wave at 0.057 Hz with adjacent palpated extension section at 0.064 Hz. MFHA shows HF activity at 0.319 Hz, twice the MFHA activity at 0.157 Hz in the palpated participant.

In Fig. 4D it appears as though palpation misses ANS activity exhibited by MFHA. However, further analyses suggest it may be a matter of detailed information, which was not graphically displayed by MFHA analyses. Figure 5A and B further demonstrate this problem by displaying two examples of different harmonic waves discarded or suppressed by MFHA analysis. Figure 5A (LF-responder) shows a dominant MFHA wave at 0.064 Hz and a smaller wave at 0.15 Hz, representing most likely an upper harmonic of the former, which is yet not displayed by MFHA. The corresponding extension section was palpated at 0.154 Hz. CRI activity computed at 0.075 Hz (≡ 4.5 cpm) was slightly off the actual MFHA at 0.064 Hz. Figure 5B shows an IM-responder with a dominant broad-based MFHA wave at 0.157 Hz and a slightly smaller narrow-based wave at 0.03 Hz (very low frequency (VLF) range), most likely a lower harmonic of the former. This, too, was discarded by MFHA. The corresponding extension section was palpated between the two peaks of MFHA and VLF activity at 0.065 Hz, and CRI activity was computed at 0.035 Hz (≡ 2.09 cpm).

Further, we assessed the presence of upper or lower harmonic waves in wavelet amplitude spectra (WAS). In both LF-responders and IM-responders, harmonic waves were present in 98% of cases in dominating VLF, LF, and IM activity. While frequencies in both examples were widely similar, the mismatch between Fig. 5A and B is striking. In the first example (Fig. 5A) palpation captured a smaller IM band wave, and the CRI corresponded with the larger LF wave. In the second example, a large IM band wave was apparently missed by palpation (interval #9) while the CRI corresponded with VLF activity. Quantification of palpated frequency bands showed that 12.1% of all palpations in IM-responders were within the VLF range (below 0.05 Hz) whereas in LF-responders only 3.4% of all palpations were VLF frequencies.

**Figure 6:**
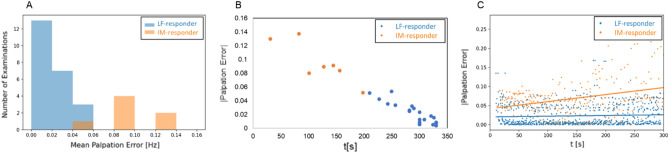
(**A**): Histogram of deviations of palpated frequencies from momentary frequency of highest amplitude (MFHA), showing a low deviation in cranial vault hold (CVH) lower harmonic waves in LF-responders (blue) and a large deviation in IM-responders (primary IM band, orange). X-axis: deviation [in Hz], y-axis: number of examinations. (**B**) Scatterplot of deviation of palpation from MFHA and low frequency (LF) interval durations during CVH. Stable extended duration of LF MFHA was related to low deviation of palpation from MFHA. (**C**) Scatterplot showing accuracy of palpation in LF-responders (blue) and IM-responders (orange) throughout CVH intervention. Plot shows a constant low error for palpation in LF-responders (horizontal blue line) and increasing error for palpation in IM-responders (ascending orange line).

A more precise estimate of deviations of palpations from MFHA was obtained by computing the root mean square of differences between MFHA and CRI (Fig. 6A–C). There were low deviations in LF-responders and high deviations in IM-responders. The palpation deviation was also highly correlated with LF interval durations (*r* = − 0.97, *p* < 0.001). This suggests that a stable strong MFHA frequency in the LF range was related to palpations with low deviation from MFHA, i.e., a relatively exact palpation. However, MFHA fluctuating between the LF, IM and HF range was related to a larger deviation to palpations (Fig. 6B). LF-responders showed relatively small deviations of palpation and MFHA across CVH intervention whereas IM-responders showed an increasing palpation error as experimental time progressed (Fig. 6C).

### Analysis of palpation phase

**Figure 7:**
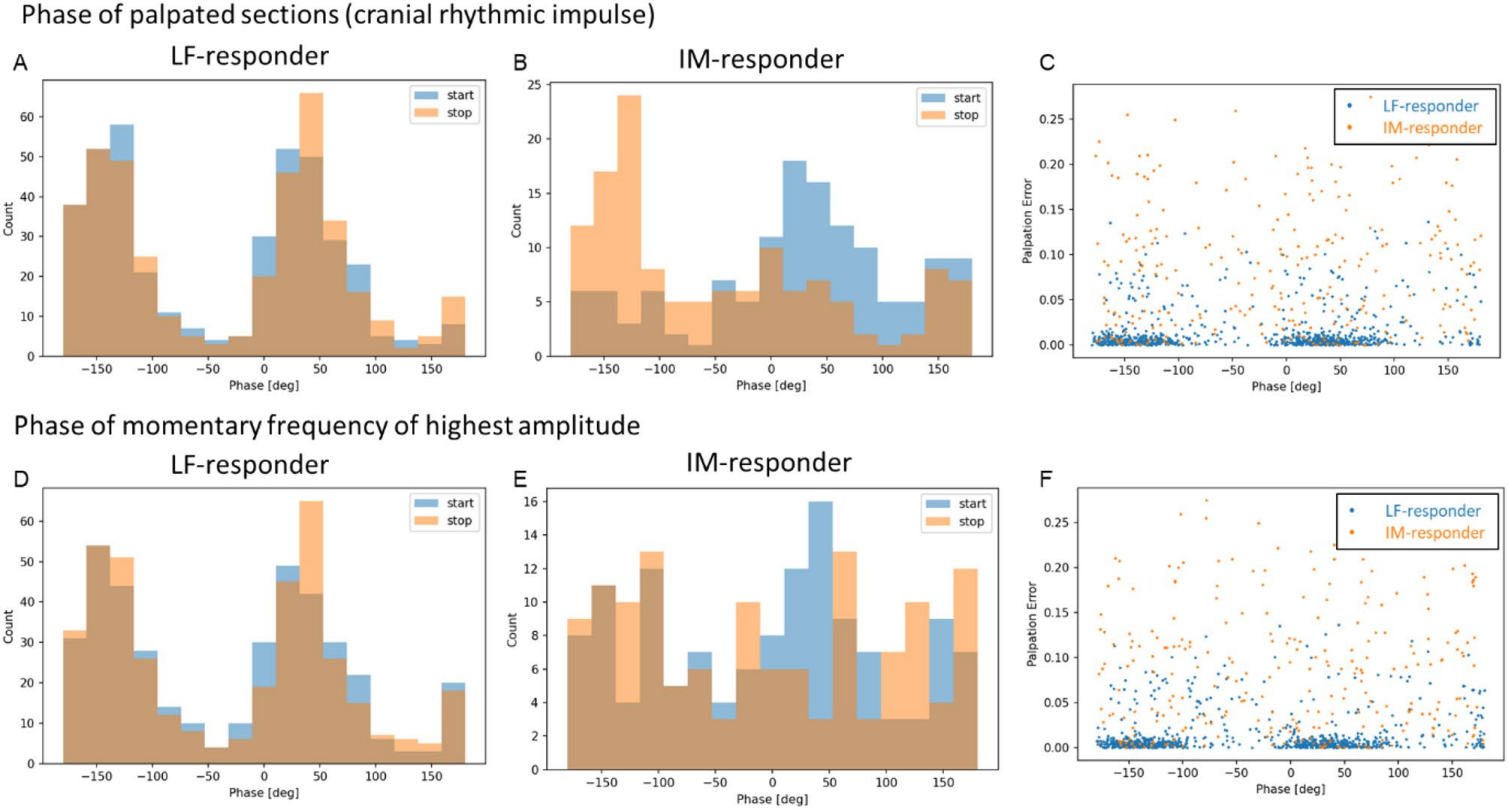
Plots of phases of palpated sections (**A**–**C**) and of phases of momentary frequency of highest amplitude (**D**–**F**) for LF-responders and IM-responders. Palpation phases were based on rates of the cranial rhythmic impulse (CRI) which were computed by multiplying durations of palpated extension sections by a factor two. (**A**) Histogram of phases of palpated sections (brown) of LF-responders for the CRI during press (start, blue) and release (stop, orange) of footswitch operation showing two separate distinct peaks at approx. − 140 and 40 degrees for both start and stop. (**B**) Histogram of phases of palpated sections (brown) of IM-responders for the CRI during press (start, blue) and release (stop, orange) of footswitch showing distinct peaks with greater overlap at − 150 degrees for stop and at 30 degrees for start (sum: 180°) yet scattered phase prevalence. (**C**) Palpation error (MFHA–CRI frequency for each palpated interval) plotted against phase of footswitch marked palpated CRI frequency. Constant low errors indicated by agglomeration in LF-responders at approx. − 150 degrees and 40 degrees. Higher randomly distributed errors at for IM-responders. (**D**) Histogram of phases of MFHA during start (blue) and stop (orange) of footswitch operation in LF-responders showing two distinct peaks at approx. − 140 and 40 degrees for both start and stop, similar to phases of palpated frequencies. (**E**) Histogram of phases of MFHA during start (blue) and stop (orange) of footswitch operation in IM-responders showing two distinct peaks at − 150 degrees for stop and at 30 degrees for start (sum: 180°), showing a scattered phase prevalence as shown for palpation in IM-responders. (**F**) Palpation error (MFHA–CRI frequency for each palpated interval) plotted against phase of computed MFHA. Constant low errors indicated by clustering in LF-responders at approx. − 150 degrees and 40 degrees. Higher randomly distributed errors for IM-responders.

Exploratory analyses of phases of palpated frequencies and MFHA at the time of push (start) and release (stop) of the footswitch were performed to rule out the possibility of ‘frequency- or rhythm-bias’, i.e., that examiners could have followed own rhythms or a certain expected rhythm without responding to real palpated sensations (Fig. 7A–F). Distinct peaks for start and stop phases of palpated frequencies and almost identical peaks for the phases of MFHA suggest that palpation occurred not arbitrarily or randomly. Both histograms of LF-responders show peaks at approx. − 140 degrees and 40 degrees for both start and stop. Histograms of phases of palpated frequencies index that the footswitch was pressed and released at approx. the lowest point and shortly after the highest point of the wave. The equal probability of each phase for start and stop indicates that extension could either be a correlate of the rising (− 180° to 0°) or falling (0°–180°) edge of the PPG signal.

**Figure 8:**
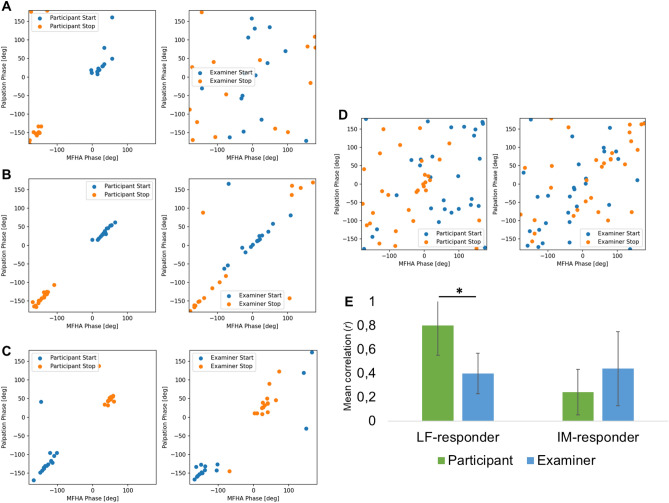
Comparison of start and stop of footswitch phases of palpated cranial rhythmic impulse (CRI, y-axis) with start and stop of phases of momentary frequency of highest amplitude (MFHA, x-axis) in participants (left columns) and examiners (right columns) for LF-responders (**A**–**C**) and IM-responders (**D**). (**A**) In most LF-responders (N = 14) start and stop of phases of CRI and MFHA responded mostly linearly in participants, and randomly in examiners. (**B**) In a smaller group of LF-responders (N = 7), start and stop of phases of CRI and MFHA in participants were linearly as in Fig. 8A. Start and stop of phases of CRI and MFHA in examiners were also linearly, yet with some outliers. Here, stop phases were at approx. − 130°, and start phases approx. + 50°. This may reflect synchronization between participants and examiners. (**C**) Fewer LF-responders (N = 2) showed comparable distribution of start and stop of phases of CRI and MFHA in participants and examiners, but at reversed order of start and stop phases as those shown in Fig. 8A and 8B. This may correspond to differences in palpated sensations of extension and flexion. (**D**) In IM-responder (N = 7), start and stop of phases of CRI and MFHA were scattered at random in participants and examiners. (**E**) Mean Pearson’s *r* correlation coefficients for CRI frequency phase and MFHA phase in LF- and IM-responders of participants and examiners. LF-responders correlated highly in participants and significantly lower in examiners. For IM-responders, there was a low correlation for both participants and examiners. Error bars indicate standard deviations.

To determine whether the high correlation of CRI and MFHA phases of participants was arbitrary and would occur at similar probability between CRI and MFHA phases of examiners, scatterplots of CRI and MFHA phases for participants and examiners were computed (see Fig. 8). This showed random distributions in IM-responders for both participants and examiners (Fig. 8D; participants: *r* = 0.24; range − 0.1 to 0.6; examiners: *r* = 0.44; range 0.1 to 0.6). On the other hand, scatterplots of CRI and MFHA phases for LF-responders showed systematic distributions clustering around − 150° and 30° in participants (*r* = 0.80; range 0.42 to 1.0). In few cases, the negative phase was related to the examiner’s start signal and the positive phase to the stop signal, while the opposite was observed in other participants (see also Fig. 7). This also showed that more than half of examiners’ scatterplots had less consistent patterns at rather random distributions of CRI and MFHA phases (N = 14, Fig. 8A), more widespread distributions of phases (N = 7, Fig. 8B), or similarly, clustered peaks of phases as were observed in participants (N = 2, Fig. 8C). For LF-responders, the CRI and MFHA phases were significantly less correlated in examiners (*r* = 0.40; range − 0.2 to 0.93) compared to participants (*t*(22) = − 2.1, *p* < 0.05; Fig. 8E).

## Discussion

This communication was a first attempt to validate cranial rhythmic impulse (CRI) activity using digitally marked palpation of extension intervals as well as frequency and phase computations of forehead skin perfusion oscillations. Two experienced examiners marked subjective sensations during manual palpation of rhythmic events in the skin of the cranium. Palpations were related to PPG data of forehead skin perfusion. Probing physiological correlates of palpation is of relevance for several reasons. Firstly, palpation of rhythmic activity in humans has a longstanding history in medicine in general. Secondly, palpation is of crucial relevance in the practice of osteopathic manipulative treatment (OMT)^30^. Thirdly, though CRI/PRM has been the subject of numerous studies, the statement of McPartland and Mein^8^ still holds today: “The CRI phenomenon is poorly understood, and its functional origin remains unknown, despite its significance in CST (cranial-sacral treatment)”.

In a previous study^22^ , we identified autonomic nervous system (ANS) activity in the low frequency (LF) and in the intermediate (IM) frequency range in forehead skin perfusion in response to a cranial vault hold (CVH) intervention. We showed that the majority (77%) of participants responded to CVH with highly stable low oscillating activity at 0.07 Hz (LF-responders), while 23% of participants showed seemingly unstable high oscillating IM-band activity at 0.147 Hz (IM-responders). In the current communication, this data set was analyzed with respect to palpations marked by two osteopathic practitioners during CVH. Footswitch-marked palpated extension sections corresponded well with recorded slow PPG waves (Fig. 3). Mean palpated CRI rates (extension interval × 2) at 0.066 Hz for LF-responders and at 0.068 Hz for IM-responders were in line with our first hypothesis that observed CRI rates would be comparable with those previously reported for experienced osteopathic practitioners (0.08 Hz; ^24^).

Wavelet amplitude spectra revealed ANS related VLF, LF, or IM activity and upper or lower harmonic waves (integer number coupling) in 98% of LF- and IM-responders, yet with differing distributions of amplitudes (see Fig. 5A and B). For example, a dominant wave at 0.07 Hz was accompanied by an upper harmonic wave (1:2) with lower amplitude at approx. 0.15 Hz (see e.g., Fig. 5A). Of note, we observed a high correlation between the mean MFHA frequencies and the mean CRI frequencies in LF-responders and in IM-responders, which confirmed our second hypothesis. Interestingly, there was a stable 1:1 ratio in LF-responders and a stable 1:2 ratio in IM-responders (mean MFHA_LF-responders_ = 0.071 Hz; mean MFHA_IM-responders_ = 0.147 Hz). This difference of objectively recorded and subjectively assessed rhythmic activity in forehead skin perfusion of IM-responders has already been reported in the literature (for a review, see Fig. 1 in 4) for the ratio between low CRI rates and rates of Traube-Hering (TH) waves (range: 0.1–0.15 Hz) (for a review, see Fig. 1 in 4). This historic TH model, however, falls short to explain the physiological backgrounds of the 1:2 ratio. TH or TH-Mayer waves in humans are associated with a narrow frequency range between 0.1 and 0.15 Hz. Furthermore, it is unlikely that these oscillations are of the same etiology as those which have been described by these historic authors. Originally, Mayer waves have been observed at 0.05 Hz in deteriorating preparations of anesthetized spontaneously breathing rabbits^31^. Furthermore, Traube’s and later authors’ pivotal works used mostly dogs for their experiments, which may have exhibited a mix of rhythms of sympathetic and reticular origin. However, while sympathetic activity is subject of scaling, meaning that the same wave appears at higher frequencies in smaller species^32^, IM or reticular activity has been shown to emerge under comparable conditions at similar frequencies in humans and in dogs^17^. Also, if oscillations recorded by us were of sympathetic origin as those reported for blood pressure, they were to exhibit tonic, that is non-rhythmic qualities^33^. This, however, is not the case since our findings exhibit rhythmically modulated activity. Therefore, we suggest IM band activity to account for 1:2 ratios between palpated CRI and instrumentally recorded MFHA since this model incorporates lower (0.075 Hz) and upper (0.3 Hz) harmonic waves of primary IM frequencies (0.12–0.18 Hz)^17^. This approach has accumulated mounting experimental evidence over the past years^19^ and has already been discussed to account for the CRI/PRM^13,20^. Our current findings appear to corroborate this approach further.

Our fourth hypothesis of the MFHA in forehead skin rhythms containing information on physiological correlates of CRI palpations is supported by findings on the high overlap of MFHA with palpated CRI rates in LF-responders. This appears as further evidence that the two examiners palpated identical rhythmic sensations at the heads of participants. A possible palpation bias from focusing on anticipated rhythmic events was ruled out by similar CRI and MFHA phases in participants (see Fig. 7A–F). In summary, these findings support the validity of palpated CRI rates by MFHA rates in LF-responders.

Comparing phases of footswitch start and stop signals during palpation of CRI activity with phases of MFHA in participants, revealed a linear 1:1 ratio in a group of 14 LF-responders. However, a random distribution of start and stop phases of CRI and MFHA in examiners suggest that there was no phase synchronization between participants and examiners (Fig. 8A). A smaller group of LF-responders (N = 7, Fig. 8B) showed a 1:1 ratio of CRI and MFHA with all stop phases at approx. − 130° and all start phases at approx. + 50° in participants and in examiners suggesting synchronization of phases. Phase synchronization, i.e., the unidirectional adjustment of palpated CRI phases with those of MFHA should be considered here since the CRI was palpated following occurrence of ANS related oscillations reflected by MFHA^34^. In the corresponding examiners, the synchronization of start and stop of phases of CRI and MFHA was comparable. Whether there is bidirectional triggering of coordination (reversed order of start and stop phases suggests this may be the case) or phase synchronization between examiners and participants cannot be inferred from the current analyses. It is, however, of high relevance and should be probed employing synchrograms and coordigrams^35^. Similarly, a reversal of respiratory sinus-arrhythmia has previously been associated with high or moderate levels of fMRI-related anxiety^36^. Correlations between mean phases of MFHA and CRI were significantly higher in participants compared to examiners suggesting successfully triggered CVH effects. In IM-responders, however, CRI and MFHA phases showed only low correlations in participants and in examiners. This suggests there was only poor synchronization in and between participants and examiners.

### Origin of the 1:2 ratio between CRI and MFHA in IM-responders

The difference of CRI-MFHA ratios between LF-responders and IM-responders may result from the MFHA analysis. As the MFHA solely displays the frequency of highest amplitude, it discards other important frequencies of lower amplitude. Our wavelet amplitude spectra (WAS) further exemplified this shortcoming, as the presence of additional frequencies of lower amplitude could be demonstrated (see Fig. 5A–D). Therefore, our terms LF-responders and IM-responders relate only to MFHA results and should be viewed with caution in the context of palpation. WAS analyses suggest that the palpation frequency in IM-responders relates to lower amplitude harmonic waves. This is in keeping with our two examiners reporting no differences in palpation sensations between LF-responders and IM-responders. Interestingly, three of five participants that were measured twice were both LF- and IM-responders. On the one hand, MFHA differences between consecutive measurements could be suggestive of different physiological reactions. On the other hand, a 2:1 ratio of MFHA and CRI as well as similar palpation frequencies indicates similar reactions and a possible shortcoming of the MFHA analysis as lower palpable PPG oscillations were only discovered in the WAS.

IM-band activity appears to be a plausible explanation for the origin of the CRI and the PRM, respectively, since inclusion of lower and upper harmonic waves broaden its primary frequency range (0.15 Hz ± 0.03 Hz) exponentially. Associated with this feature is phase-synchronization at 1:1 or 1:2 integer number ratios with respiration, HRV, and blood pressure^17^. This might become a source of confounding as frequencies in interacting subjects synchronize and rhythmic differences 'palpably' dissolve during phase synchronizations. This notion is supported by our findings in a smaller group of LF-responders on highly synchronized phases of CRI and MFHA in both participants and examiners (see Fig. 8B). This, however, mandates further comprehensive data recording and data analyses of synchronization and coordination including at least one additional peripheral system.

### Flexion and extension: fact or fancy

Flexion and extension refer to palpation sensations in trained observers^7,10^. These terms relate to physiological correlates such as minute scalp motion strong enough to be palpated by sufficiently trained therapists^12^. Osteopaths commonly describe extension as movement towards the examiner and flexion as a movement away from the examiner. In our investigation we marked extension phases to compute the complete CRI. This supplied results which apparently support the construct of flexion and extension given our results for MFHA and WAS. However, based on our data we cannot relate phases of forehead skin MFHA to manually palpated extension and flexion, whereas extension (and flexion) was well distinguishable by examiners. Therefore, a rising or falling edge of the PPG wave is obviously not permanently related to their palpated sensations.

### Limitations

The large variation in physiological rhythms^37^ as well as in CRI rates^11,38^ is a general complication of osteopathic research. The small sample size, the small number of examiners as well as the inclusion of five participants measured on two occasions limit the generalizability of our results. Furthermore, recording and analyses of PPG signals as the only physiological system is prone to leave important physiological questions unanswered.

### Outlook

Osteopathy in the cranial field has often been criticized for its contradictory findings. This concerns foremost the limited interrater reliability since experienced and beginning osteopaths showed findings differing by a factor two. By showing overlapping variability of MFHA, WAS, palpation frequencies, and phases during CVH, our analyses offer a first glimpse into resolving the CRI’s problematic interrater reliability. However, in our data (Fig. 8C) the interaction of participant and examiner may bear potential to induce significant systemic physiological changes. Factors determining these interactions should be considered in further investigations. Furthermore, we defined two groups of participants responding to CVH in physiologically different modes. Further studies will show whether responses to CVH are related to behavioral parameters and to treatment outcome in general. This will be of importance when investigating CRI and MFHA in patients.

## Data Availability

The datasets used and/or analyzed during the current study are available from the corresponding author on reasonable request.
